# Mapping of dressed and processed poultry products in Bangladesh: Identifying the food safety risks for policy intervention

**DOI:** 10.1007/s11259-023-10153-7

**Published:** 2023-06-27

**Authors:** Jinnat Ferdous, Md Helal Uddin, Rashed Mahmud, Mathew Hennessey, Abdullah Al Sattar, Suman Das Gupta, Justine S. Gibson, Robyn Alders, Joerg Henning, Guillaume Fournié, Md. Ahasanul Hoque

**Affiliations:** 1 School of Veterinary Science, The University of Queensland, Queensland, Australia; 2 Chattogram Veterinary and Animal Sciences University, Chittagong, Bangladesh; 3 Veterinary Epidemiology, Economics and Public Health Group, Department of Pathobiology and Population Sciences, Royal Veterinary College, London, UK; 4 Gulbali Institute, Charles Sturt University, Wagga Wagga, NSW Australia; 5 Development Policy Centre, Australian National University, Canberra, NSW Australia; 6 Global Health Program, Chatham House, London, UK; 7 INRAE, VetAgro Sup, UMR EPIA, Université de Lyon, Marcy l’Etoile, 69280 France; 8 INRAE, VetAgro Sup, UMR EPIA, Université Clermont Auvergne, Saint Genes Champanelle, 63122 France

**Keywords:** Food-borne pathogens, Food safety, Live bird market, Poultry, Zoonosis

## Abstract

Bangladesh’s commercial poultry production is growing rapidly, including the commercial processing of poultry. This expansion of poultry processing plants is fueled by the belief that this sub-sector provides safer food and has less food-borne disease risks compared to traditional live bird markets (LBMs). The purpose of this study is to describe Bangladesh’s dressed and processed poultry production and distribution network (PDN), identify what and where quality control occurs, and suggest where improvements could be made. Engaging with PDN for dressed and processed poultry, we used in-depth interviews with key informants to identify the stakeholders involved and their connections with other poultry PDNs. In addition, we mapped out the supply and distribution of dressed and processed poultry and quality control processes occurring throughout the network. We argue that dressed and processed poultry PDNs are closely connected with traditional PDNs such as LBMs, with multiple crossover points between them. Also, there is a lack of consistency in quality control testing and a lack of meat traceability. Consequently, perceptions of dressed and processed poultry being safer than birds from LBMs needs to be treated with caution. Otherwise, unsubstantiated consumer confidence in dressed poultry may inadvertently increase the risk of food-borne diseases from these products.

**Supplementary Information:**

The online version contains supplementary material available at 10.1007/s11259-023-10153-7.

## Introduction

Approximately a quarter of all animal proteins consumed by humans in Bangladesh are obtained from poultry (Hamid et al. 2017). Poultry consumption per capita per year is estimated to be 6.3–8.5 kg (LightCastle 2020; WPSA 2020) and is expected to increase by 17% by 2025 (Statista 2022) . While fish protein (19.7 kg) is still the most consumed animal protein in Bangladesh, exotic broiler chicken meat is the most common poultry type consumed per capita per year (5.5 kg), and small portions attributed to Sonali chicken (1.7 kg), duck (0.4 kg) and other poultry species (0.9 kg) (LightCastle 2020; Hennessey et al. 2021). The phenotypic homogeneity of exotic broiler chickens such as Hubbard Classic, Cobb-500, Ross, and Hybro-PN (Tan et al. 2021) has facilitated the global development of standardized and automatized processing plants (Owens et al. 2001) , with this growth being fostered by advances in food science, food microbiology, engineering, and marketing (EFSA/ECDC 2019). Sonali chickens are a crossbred variety between Rhode Island Red males and Fayoumi females introduced to Bangladesh in the 1990s as part of the Department of Livestock Services’ (DLS) rural poultry program (Howlader et al. 2022) and an increasingly common variety to produce (Hennessey et al. 2021). Most poultry meat in Bangladesh is produced on commercial poultry farms (60%) with the rest occurring on traditional backyard farms (LightCastle 2020). Thus, through the provision of key proteins, micronutrients, and employment, poultry production contributes to Bangladesh achieving multiple Sustainable Development Goals (SDGs)^1^ (UN 2023).

Despite the highly pathogenic avian influenza H5N1 outbreak in 2007 causing significant disruption to the poultry sector, the broiler and layer industry in Bangladesh have experienced annual growth of 3% and 5%, respectively over recent decades (FAOSTAT 2021; Hamid et al. 2017). Consequently, the sector employs almost eight million people in Bangladesh (Das et al. 2008; USDA 2019). In 2019, the total poultry population (at any one time) in Bangladesh was estimated to be in the region of 400 million birds (Department of Livestock Services (DLS), 2020; Hennessey et al. 2021), involving around 70,000 commercial chicken grower farms throughout the country. The annual production of broiler birds is estimated to be more than half a billion (Hennessey et al. 2021). These, along with layer birds, are supported by 16 grandparent farms, 206 parent farms and 198 registered feed mills producing around 5.5 million metric tons of feed annually (WPSA 2020).

Most poultry, including exotic broiler chickens, are supplied to consumers in Bangladesh via live bird markets (LBMs) (Moyen et al. 2021) where birds are slaughtered and processed on demand at the point of sale. Bangladesh’s first commercial company-owned slaughterhouse became operational in 1998. Development of this sub-sector remained slow over the following decade until the establishment of two additional companies in 2009 and six more thereafter, distributed across different regions of the country. Recently, news outlets have reported that consumer demand for dressed and processed poultry products is increasing due to growing concerns over meat hygiene and safety in LBMs (LightCastle 2020).

However, the configurations of the PDN of dressed poultry and processed poultry products are unknown. Additionally, despite poultry meat being a major source of food-borne pathogens, including *Campylobacter* spp. and *Salmonella* spp. (EFSA/ECDC 2019), the assessment of meat quality and safety control procedures and its certification by governmental authorities (such as the Bangladesh Food Safety Authority (BFSA), and Bangladesh Standards and Testing Institution (BSTI)) has not been described. Therefore, we aimed to address this knowledge gap through the following objectives: 1) to map the PDN of dressed poultry and processed poultry products in Bangladesh and 2) to assess quality control procedures and hygiene practices that could impact contamination of poultry products by food-borne pathogens.

## Materials and Methods

### Data Collection

#### Poultry slaughtering and processing companies

Through consultations with stakeholders (e.g., representatives from the poultry industry, university, livestock research institute, and DLS office) who are knowledgeable about the poultry production systems in Bangladesh, all companies owning poultry slaughterhouses and/or processing plants in Bangladesh were identified (*n* = 11). All eleven companies were contacted, and nine agreed to participate in the study. To map the PDN of dressed poultry and processed poultry products, in-depth key informant interviews with company representatives, managers, and directors were conducted from June to September 2020. An interview guide for slaughterhouses (Supplementary Information 1) was developed and covered the following topics: i) sources of poultry, ii) modes of transport, iii) slaughtering and processing practices, iv) quality control and hygiene practices in processing plants and during transportation, and v) distribution of finished poultry products.

For our study, slaughterhouses were defined as “premises, including animal transport facilities, used for the slaughter of animals for human consumption or for animal feeding and approved by the national veterinary services or other competent authorities” (Shimshony and Chaudry 2005). “Dressed poultry” referred to dressed whole poultry, while “processed poultry products” referred to poultry that have undergone processing such as being cut into specific sizes, shapes, or portions; or marinated with spices. “Contracted farms” referred to farms which were provided with production inputs (day-old chicks, feed, and possibly medicine) by a company and are committed by a written contract to sell their chickens at defined rate to that company at the end of each production cycle. “Credit-based farms” referred to farms which were provided production inputs on credit by feed and chick dealers, who generally facilitated the sale of chickens at the end of the production cycle, from which the credits were reimbursed. In contrast, “independent farms” did not depend on the aforementioned arrangements to obtain production inputs and were free to sell to anyone, but also sell through feed dealers.

#### Supermarkets

Supermarkets, where dressed poultry and processed poultry products were sold to consumers, were also surveyed. All supermarket outlets in Dhaka and Chattogram, the two most populated cities in Bangladesh, were identified through an online search and contacted over email and phone. In Dhaka city, we identified 90 outlets, belonging to ten different supermarket chains. However, only the manager from one company – which operated the majority of outlets in Dhaka—agreed to participate. In Chattogram city, there were three supermarket companies (one with two outlets, two with one outlet each) and four individually owned supermarkets selling dressed or live poultry or both. Here, five supermarket managers agreed to participate (one with two outlets, and four with one outlet each).

An interview guide for supermarkets (Supplementary Information 2) was developed covering the following topics: i) types of poultry sold in supermarkets, ii) sources of poultry, iii) quality control and hygiene practices, iv) types of customers and v) modes of delivery to consumers.

For all interviews, open ended questions were used to reduce interviewer confirmation bias and interviewee social desirability bias (Powell 2013). Informed verbal consent was obtained prior to the interview, and interviews were voice recorded where consent allowed. Interviews lasted between 40 to 60 min, and recorded in Bengali, and later transcribed into English by the primary author.

### Data Analysis

The study was done using deductive thematic analysis described by (Chan et al. 2015). The first author familiarized herself with the raw data by listening to the interviews and reviewing interview scripts to enlist main ideas. Then she identified the main themes and sub-themes, extracted the data using a separate coding template in Microsoft Word (version 365, 2010) for each poultry company with slaughterhouses and/or processing plants and for each supermarket. After coding, rearranging was done according to thematic framework, and finally mapping and interpretation was done based on the research objectives (Pope et al. 2000) .

## Results

### Poultry slaughtering and processing companies

**Fig. 1:**
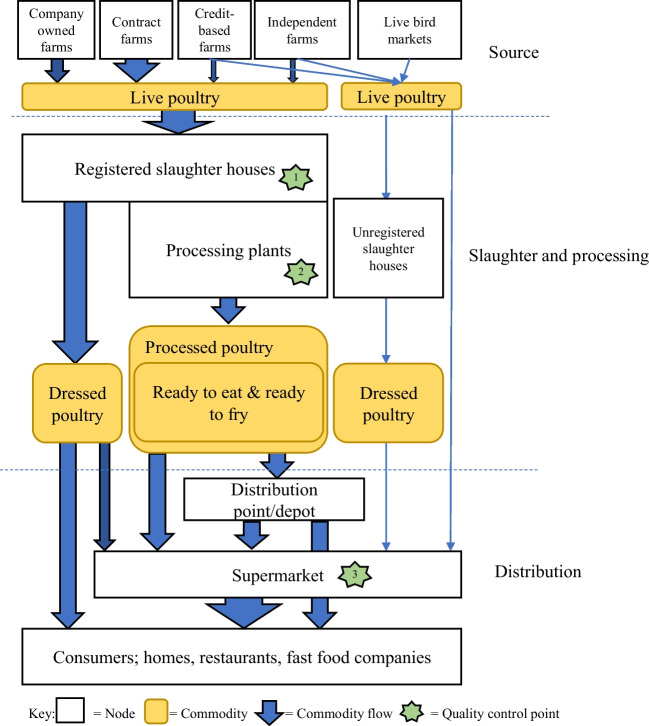
Production and Distribution Networks (PDN) of dressed poultry and processed poultry products in Bangladesh. The size of the arrows indicates the magnitude of poultry numbers in the part of the chain

The nine interviewed companies with established slaughterhouses processed around 50,000 birds a day, equating to around 3% of the total broiler produced per day in Bangladesh (Table 1). All nine companies participating in the study have head offices in Dhaka but had slaughterhouses and processing plants distributed across different districts of Bangladesh. Seven companies operated their own commercial poultry farms, with the other two companies only sourcing poultry other than raising them themselves. Around 90% of poultry were supplied to company slaughterhouses from their own commercial farms, credit-based farms, and contracted farms. The remaining 10% came from independent farms and traders, small-scale farms of which poultry production was supported, and sales facilitated by non-governmental organizations (Fig. 1).

**Table 1:** Participant company features and their source of poultry

			Source of poultry				
Company	Species slaughtered and dressed	Number dressed /day	Own farms	Dealers’ Credit farms	Contract farms	Independent farms	Approved supplier/ dealer
1	Broiler and Sonali	3500–4000	35–40%	25–30%	30–35%	-	5–10%
2	Broiler (90%) and Sonali (10%)	2000	20%	-	70–80%	Occasionally	-
3	Broiler	8000–10000	70%	-	30%	-	-
4	Broiler	5000	20–30%	-	70–80%	-	-
5	Broiler	5000	100%	-		-	-
6	Broiler	9000	100%	-		-	-
7	Broiler	5000–6000	70%	-	30%	-	-
8	Broiler (90-95%) and others	6500	No farm of their own	-	100% (Broiler)	Other	Other
9	Broiler (90-955%) and others	3000–6000	No farm of their own	-	70–80%	-	20–30%

Poultry slaughter by the nine companies ranged from 2,000 to 9,000 birds per day. Five companies slaughtered only exotic broiler chickens, two companies slaughtered exotic broiler (90%) and Sonali chickens (10%), while the other two slaughtered multiple chicken types and poultry species including Sonali and indigenous chickens, pigeons, ducks, geese, quails, turkeys, though exotic broiler accounted for the majority (90–95%).

In all slaughterhouses, exotic broiler chickens were slaughtered and processed using a semi-automatic system. Hanging of birds, slaughtering, evisceration, grading, portion cutting, and packaging were done manually. Weighing, stunning, scalding, head removal, defeathering, neck and hock cutting, and chilling were automatized. For other chicken types and poultry species, slaughtering was entirely manual:

“All the slaughtering companies in Bangladesh have semi-automatic processes mainly. The step where we need to cut into different portions, that’s when human intervention is needed, but before that human handling is not necessary actually” (Director of slaughtering company 5).

At all sites, slaughtering was supervised by a prayer leader of a mosque, known as Imam, employed by the company to ensure compliance with Halal standards (Shahdan et al. 2016).

**Fig. 2:**
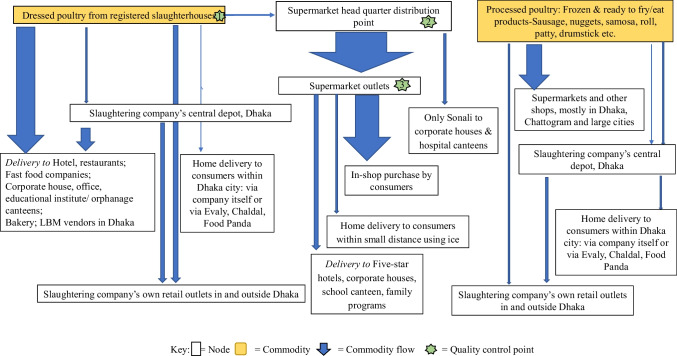
Types of consumer end points for dressed poultry and processed poultry products in Bangladesh

Eight companies owned processing plants; seven were located adjacent to the slaughterhouses and one was in a different location to the slaughterhouse. Processing plants used meat from dressed poultry to produce ready-to-fry (e.g., chicken rolls, samosas, nuggets) and ready-to-eat (e.g., chicken sausage) products. Dressed and processed poultry products were then stored in processing plants in a chiller (0–4° C) or freezer room (-18 to -20° C) depending on the product requirements until distributed. Products were then distributed by companies to different sales points via chilled and/or freezer vans, mainly within Dhaka city. The delivery of products from sales points to individual consumers’ homes was an option offered by five companies, four of which had contracts with food delivery companies. However, this accounted for less than 10% of the products sold by these companies (Fig. 2).

#### Hygiene and quality control points

In all slaughterhouses, ante-mortem, and post-mortem examination of randomly selected birds from each batch (i.e., birds from the same farm) were conducted by company employed veterinarians to ensure quality control (Table 2). There was no set requirement for the number and selection of birds to be examined, but instead, this was determined by the veterinarian dependent on whether clinical signs were observed. Seven companies had their own laboratory facilities, one company used an external diagnostic laboratory, and one company currently does not have a laboratory, though had expressed a desire to address this deficit:

“*We do not have any laboratory of our own at this moment, but we have taken all the preparation to establish one soon*.” (Manager of slaughtering company 4)

**Table 2:**
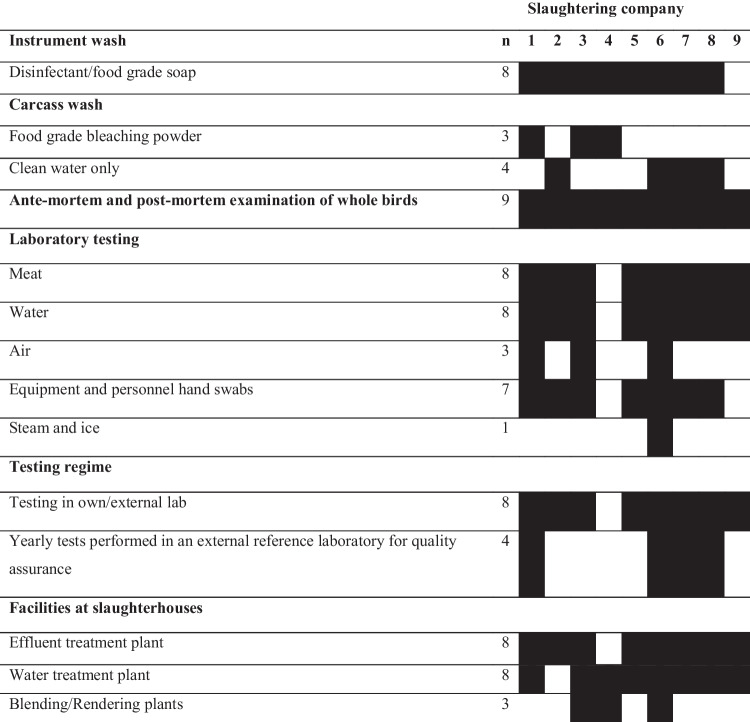
Food safety measures practiced in nine slaughtering companies

^a^Darker cells represent practicing; white cells represent not practicing.

Equipment was cleaned and then disinfected with bleaching powder (Calcium hypochlorite) in eight slaughterhouses once to twice a day before and/or after business hours. Three slaughterhouses washed the dressed birds with food grade bleaching powder dissolved in water, whereas four companies washed the dressed birds with clean water only. Effluent and water treatment plants were present in eight of the slaughterhouses and processing plants.

Eight of the companies performed microbiological sampling of meat looking for total viable count (TVC) and microorganisms such as *Escherichia coli, Salmonella* spp., *Staphylococcus aureus*, *Listeria* spp*.*, yeasts and fungi. The frequency of testing varied between companies, from weekly to bimonthly, and consisted of ten samples (100 g meat and skin) randomly collected from each batch of birds (Table 3). Water (*n* = 8), air (*n* = 3), equipment and personnel hand swabs (*n* = 7), steam and ice (*n* = 1) samples were collected and tested once monthly, bi-weekly or weekly for microbiological testing as above, and pH, water hardness, and chlorine, arsenic, and iron levels were tested; though testing varied between companies and sample types. Some companies (*n* = 4) also conducted these analyses annually using an external laboratory for validation. Antimicrobial residue testing was conducted annually for a limited set of antimicrobial classes by four companies through external laboratories (Table 3).

**Table 3:** Microbiological testing done by slaughtering companies (*N* = 9; one company did not perform any testing) on a regular basis

	Number of companies performing the test				
Test for	Meat	Water	Air	Instruments and personnel hand swab	Steam and ice
*Escherichia coli*	8	6	1	6	1
*Salmonella* spp.	8	6	1	6	1
*Staphylococcus aureus*	5	3	1	3	1
*Listeria* spp.	2	1	1	1	1
Coliform	6	4	1	3	1
Yeast/mold	3	1	2	2	
Total viable count (TVC)	8	7	2	7	1
Antimicrobial residues	4				
Heavy metal	1	3			1
pH		1			

Two companies also collected samples from their supplying farms twice a year, to test for the presence of antimicrobial residues:

“*We test meat samples for antimicrobial residues and heavy metals monthly once, but we do not test instruments or personnel hand swab samples. However, if any of the personnel in the slaughterhouse have any cold symptoms, we do regular health checkups of that person*” (Director of slaughtering company 9)

Companies reported that their customers – supermarkets, restaurants, and fast-food companies required testing for *Escherichia coli, Salmonella* spp., and *Staphylococcus aureus*. None of the companies attempted to detect contamination of meat by *Campylobacter* spp. Due to the time taken to complete testing (an average of 5 days), products which were contaminated/unsuitable for consumption could not be recalled. No testing was performed in downstream nodes (i.e., company distribution or sales points). However, companies maintained records which could be useful for identifying contaminated batches and the supplying farms. But in practice, contaminated batches were not traced back to their supplying farms. If any sample tested positive, then company officers reported that they would reinforce the hygiene and sanitation of their slaughterhouse.

### Supermarkets

It was estimated that supermarkets in Dhaka (48/90) and Chattogram (5/7) sold an average of 4000 kg and 73 kg of dressed poultry meat per day, respectively, equating to less than 1% of the average daily total amount of poultry meat produced in Bangladesh (DLS 2020). This is likely to be an underestimation given that there were many smaller, privately-owned supermarkets offering dressed poultry for sale in other cities that were not accounted for here. Further quantification would therefore require additional investigation.

#### Dhaka

Most dressed poultry sold by supermarkets were procured from live bird markets through middlemen rather than directly from the company run slaughterhouses. Indeed, only one supermarket company collected dressed exotic broiler chickens from an established poultry slaughtering company (Fig. 1):

“*As we collect dressed broiler chickens from a reputed poultry slaughtering company, we believe its quality being exactly what we want. They will not provide us defective chickens*.” (Head of distribution point of the supermarket)

This company, the largest supermarket company in Dhaka, purchased between 3,000–3,500 kg of dressed exotic broiler chickens per day from a poultry company slaughterhouse. Dressed poultry were transported by the poultry company’s own cold storage van from the slaughterhouse to the supermarket company’s distribution point. Other chicken types (Sonali and indigenous/deshi) and duck, geese, quail, pigeon, and turkey were sourced from middlemen/dealers. These birds were transported live by middlemen/dealers from farms to their own informal, non-registered slaughterhouses. There were four such slaughterhouses located in Dhaka, from where dressed poultry were collected by the supermarket company’s own cold storage van and brought to the distribution point. However, some live Sonali and deshi chickens were also brought by middlemen/dealers from farms to the supermarket company’s distribution point.

“*Mostly people like to buy live ones and take them as it is from the outlet. But sometimes if any customer wants, then we slaughter the birds for them*” (Head of distribution point of the supermarket)

These live chickens accounted for < 5% of poultry marketed by the company. Dressed and live poultry were distributed separately, the former using a cold storage van, to all companies’ outlets in Dhaka (Fig. 1).

#### Chattogram

Supermarkets in Chattogram mostly collect dressed poultry from local live bird markets or middlemen. These middlemen sold poultry from their own farms, sourced poultry from other farms, and slaughtered them in their own informal, non-registered slaughterhouses. The reason for not sourcing dressed birds from slaughtering companies was stated as financial:

“*If we source dressed birds from slaughtering companies, the cost will be higher than collecting from other sources. Customers will not be happy to pay that high price and it will not be possible for us to get profit from the sale*.” (Head of distribution point of the supermarket)

Four out of the five supermarket companies in the study brought both live and dressed birds to their outlets. Only one company was found to offer live deshi, Sonali, and spent hens for sale in their outlets. Though the other companies also brought live birds into their outlets, these birds were generally slaughtered and dressed upon their arrival, except for when an order for live birds had been received. These live birds would be then slaughtered within a day in the presence of the customer. Different chicken types (exotic broiler, Sonali, deshi, spent hens) and duck, quail, pigeon, and turkey were sold in supermarket outlets in Chattogram. Customers were mainly consumers, and hotels, cafeterias, corporate houses, or family programs like weddings, birthday parties, etc. All but one outlet received poultry and their products daily, with the remaining outlet only being supplied after receiving consumers’ orders. Four of the supermarket companies offered a home delivery service in Chattogram (Fig. 2).

#### Hygiene and quality control

*Dhaka*- Supermarket companies did not subject farms from which they sourced poultry and unregistered slaughter places to controls or regular laboratory diagnostic testing for pathogens, residues, or heavy metals. Moreover, intermediaries/suppliers did not have defined procedures for cleaning and disinfection of their unregistered slaughterhouses and their vehicles. However, the head of distribution for the supermarket chain in Dhaka explained how their staff conduct poultry farm inspections:

“*We have specific suppliers for poultry who collect poultry from farmers. But our quality control staff visits the farms monthly once/twice to check the farm’s condition*” (Head of distribution point of the supermarket).

Usually, the dressed and live poultry were examined for bruising, blood spots, and breakages in legs and/or wings (i.e., visual appearance) by the personnel in the central distribution point in Dhaka. Products that appeared damaged or did not pass organoleptic tests were returned to their respective sources or suppliers. Quality control personnel were not veterinarians but lay staff who relied on their experience to assess poultry quality. Distribution outlets also examined products on receipt and if signs of damage or deterioration were observed, then they were returned to the central distribution point. Dressed meat was displayed in outlets in a chiller at 4^0^C for 36 h after which it was discarded. In supermarket outlets, live poultry were often kept in glass cages in close proximity to where dressed poultry was kept for sale. Though these cages had ventilation pipes connected to the outside of the shop, live birds were handled by the same staff who handled dressed poultry.

*Chattogram*- The same practices regarding the source farms’ biosecurity, laboratory tests for pathogens or heavy metals or residues, and cleanliness and hygiene conditions of the transport vehicles were also reported for supermarkets. Birds were sometimes selected by outlet employees before slaughtering when they were sourced from and slaughtered in live bird markets. Dressed poultry were washed with water in supermarket outlets (*n* = 3) or by the middleman (*n* = 2) in Chattogram. Only three supermarket companies used ice during dressed poultry transportation from source to outlets. One supermarket company required that dressed poultry were brought to the outlet within an hour of slaughtering where staff could inspect the condition of the carcasses to ensure quality control.

The supermarkets mainly depended on visual appearance (colour, texture, and smell) rather than doing any microbiological testing by their own or by the sources, as the sources were mostly middlemen and live bird markets. Only one supermarket conducted post-mortem examinations by its quality control staff if there was suspicion about the health status of the purchased birds. If they found any abnormalities after the post-mortem, they condemned the meat without any laboratory testing. The display shelves were sanitized with chlorhexidine gluconate before dressed meats were displayed. Dressed meat was displayed in a chiller at 4^0^C for varying hours (36–72 h) after which unsold meat was supplied to fish farms (*n* = 2) or returned to their suppliers (*n* = 3). Only three supermarkets used ice during transport from the outlet to consumers’ houses when there was home delivery.

In outlets where live birds were kept and slaughtered in the presence of customers, the butcher section was cleaned and disinfected with bleaching powder or chlorhexidine gluconate at the start of each day. Equipment and the butchering area were also cleaned and disinfected immediately after slaughter. Waste was stored in bins and collected daily by the city’s local government services. All personnel were reported to wear aprons, masks, and gloves.

## Discussion

Dressed poultry and processed poultry product production is a growing sector in Bangladesh, fueled by consumer desire for food safety and a perception that these products are safer than traditional poultry. However, we identified several risky practices and varying levels of quality checks and controls among stakeholders – poultry companies, slaughterhouses, and supermarkets – involved in producing dressed and processed poultry, which lead us to question this perception of safety.

### Poultry companies owning slaughterhouses

Most companies owning slaughterhouses have their own managed poultry farms, which may facilitate traceability and the enforcement of desirable production standards. However, when poultry are supplied by sources other than their own farms, it may be more difficult for companies to ensure such standards. Concerning, small-to medium scale broiler farms in Bangladesh have been found to have inadequate biosecurity (Imam et al. 2021). Moreover, in a study assessing antimicrobial use in Bangladeshi poultry farms, 84.7% of farmers were found to use antimicrobials prophylactically and 36.1% of broiler farms were found to have sold chickens without maintaining appropriate antimicrobial withdrawal period (Imam et al. 2020). This high level of antimicrobial usage is concerning, with some authors suggesting that farmers have insufficient knowledge about antimicrobial usage and withdrawal periods (Hassan et al. 2021). Likewise, when poultry are supplied by dealers, the companies cannot control the conditions under which birds are transported to slaughterhouses. Yet, incentives and or motivations are low for middlemen to adopt time-consuming and costly hygienic measures. However, hygienic conditions of poultry transport vehicles belonging big middlemen are relatively better (Personal observation, MA Hoque).

Most high-income countries (HIC) have standards for poultry meat production and processing and these standards are enforced by federal and state governments in these HIC (FoodStandards 2019). However, such standards have not been developed for poultry meat processing companies in Bangladesh. These companies are supposed to follow the World Poultry Science Association (WPSA) standards or other international slaughtering standards (LightCastle 2020). But BSTI is the authority who gives the certificate for quality of dressed and/or processed poultry products. Moreover, International Organisation for Standardization (ISO) have their own agent in Bangladesh who give ISO certificate by inspection of slaughtering and processing plants. Moreover, BFSA with the help of Islamic Foundation also visit the slaughtering and processing plants and give the Halal certificate by sudden visits. And recently the BFSA has got the authority to issue health certificates of products which can be imported abroad.

Bangladesh has legal framework to ensure the quality of food produced by the companies. According to Food Safety Act 2013, if anyone sells/stores/produces sick or dead birds, the person will be punished with one to three years of jail time (lowest 1 year) or 0.3 – 0.6 million BDT fine payments. There is a separate court, called as Food Court, for the enforcement of Food Safety Act 2013. The judicial power of the court is either in the hand of judicial magistrate or metropolitan magistrate. On the other hand, if there is any offense regarding slaughter (such as poor slaughtering environment, sick or diseased slaughter personnel, poor waste management etc.), that falls under Slaughter Act 2011. The punishment for breaching the Slaughter Act 2011 is, one-year jailtime or 5000 to 25,000 BDT fine payments or both. A veterinarian (appointed by the Directorate General -Livestock) will punish them under Mobile Court Act 2009. Thus, it is therefore highly recommended that the WPSA Bangladesh Branch, DLS, BSTI and BFSA establish guidelines for the slaughtering and processing of poultry, including setting up policies for monitoring adherence and the enforcement of these guidelines.

Laboratory diagnostic test protocols are not harmonized across the companies in Bangladesh. While most companies regularly tested their products for a range of microorganisms, this did not include *Campylobacter* spp. In contrast, HIC such as Australia and New Zealand, follow the Primary Production and Processing (PPP) Standard for poultry meat, aiming to lower the prevalence of *Campylobacter* and *Salmonella* as these are the two most common bacteria present in raw poultry meat causing illness in humans (EFSA/ECDC 2019; FoodStandards 2019). Therefore, screening for *Campylobacter* should be included in the regular laboratory testing protocols of poultry processing companies in Bangladesh. The One Health Poultry Hub Bangladesh has optimized the standard operating procedure (SOP) for *Campylobacter* testing in Bangladesh, which has been implemented in laboratories of the Poultry Research & Training Center (PRTC), CVASU and Bangladesh Livestock Research Institute (BLRI). These SOPs should be adopted also in poultry processing companies’ laboratories in Bangladesh.

All companies should also test their products for antimicrobial residues and heavy metals. At present, test results are not generated rapidly enough to recall contaminated products. More advanced molecular techniques, such as High-Performance Liquid Chromatography (HPLC) or Enzyme-Linked Immunosorbent Assay (ELISA), should be considered for antimicrobial residue and heavy metal testing to reduce the time between sampling and the generation of test results (Hoelzer et al. 2018). Companies should also not just focus on their slaughterhouse’s hygiene but also trace back flocks with elevated antimicrobial residues and heavy metal levels. This would allow to reduce the application of antimicrobials on poultry farms and to reduce exposure of birds to heavy metals, and thereby mitigate the prevalence of health hazards at the production stage.

The companies did not use data on the occurrence of health hazards to identify temporal trends. Yet, this data could inform risk assessments and the implementation of risk mitigation interventions along the networks. Companies should also implement disease surveillance activities on the farms supplying them, especially if they consider exporting poultry meat in the future.

### Supermarkets

As supermarket companies were mainly supplied through LBMs and middlemen, the actual origins of the birds they were supplied with were generally unknown, and they were unable to monitor and control farm biosecurity standards, antimicrobial usage, and the hygienic and sanitary conditions under which poultry were transported, marketed, and slaughtered. Hygiene standards are usually low in LBMs (Chowdhury et al. 2020), with the same bucket of water being used to wash multiple bird carcasses, posing a risk of bacterial cross contamination. *Salmonella* contamination in dressed poultry and poultry products has been commonly reported in low-middle income countries (LMIC) (Khan et al. 2018; Mpundu et al. 2019). For instance, in Trinidad, (Khan et al. 2018) detected *Salmonella* spp. in chicken carcasses in 20.5% and 8.3% of retail shops and supermarkets, respectively. *E. coli* and *Salmonella* were detected in 70% and 2.5% of dressed chickens in retail outlets in Zambia, respectively; with higher levels of contamination in washed carcasses compared to pre-washed carcasses (Mpundu et al. 2019). *Campylobacter* spp. were isolated from 54–75% broiler meat samples from LBMs of several districts of Bangladesh (Kabir et al. 2014; Md 2018; Neogi et al. 2020). (Uddin et al. 2019) detected *Salmonella* and *E. coli* in dressed poultry meat from supermarkets in Dhaka and suggested that the possible cause of bacterial contamination was unhygienic processing and handling of poultry and poultry products.

However, supermarket managers reported that supermarket outlets strictly enforced the usage of personal protective equipment and frequent cleaning and disinfection. While this may contribute to a reduction of the risk of cross-contamination (Luber 2009; Al Banna et al. 2021), and attempts were made to minimize contact between poultry products and live poultry – with live poultry being confined in glass cages with a separate line of ventilation – the such risk of cross-contamination remains, as both bird products and live birds are kept near each other on the same floor and are handled by the same people (de Perio et al. 2013; Rahman et al. 2020).

There was no legislation about the recruitment of veterinarians by supermarket companies. Thus, supermarket companies don’t have veterinarians in their teams, who should oversee the inspection and condemnation of dressed poultry unfit for human consumption. For instance, Australia and New Zealand’s laws state the requirement for appointing a Food Safety Supervisor, and that the staff in charge of handling food products should be trained in food safety (FoodSafety 2016).While we could not assess whether supermarket staff have received any kind of food safety training, we therefore recommend the completion of a Food Handler Course as a requirement for all supermarket staff upon their recruitment, and the harmonization of standard operating procedures related to the transport, handling, and processing of poultry.

There is a general belief among the urban consumers that dressed poultry would be safer to consume than the traditional poultry sold through LBMs in Bangladesh (Islam et al. 2017; Khairunnesa et al. 2020; LightCastle 2020). But this study showed that the dressed poultry production and distribution channels overlapped with traditional networks via LBMs. Thus, the consumer’s confidence that dressed poultry sold in supermarkets were at lower risk than poultry sold in LBMs was somewhat wrong and may increase the risk of food-borne disease from these supermarket products. Thus, as mentioned above, there is a need to enforce the present food safety legislation in Bangladesh, while awareness campaigns aimed at consumers should emphasize the necessity of proper cooking of dressed poultry and poultry products to prevent food-borne diseases (USDA 2016).

## Conclusion

This study described the PDN of dressed poultry and processed poultry products for the first time in Bangladesh. The perceptions of these commodities being safer than birds sold in LBMs may be misleading given interconnections across PDNs. The lack of legislation, and its enforcement, should be addressed by regulatory authorities. The implementation of traceability schemes and the standardization of SOPs related to quality control, slaughtering and processing of poultry should be a requirement for all slaughtering and supermarket companies.

## Research limitations

While there were more than 90 supermarket outlets in Dhaka, we were only able to recruit the one supermarket company which operated more than 50 outlets (out of these > 90) in Dhaka. However, this supermarket company is the largest in the country. Besides, this study was conducted while movement restrictions related to COVID-19 were enforced, thus we could not conduct face-to-face interviews, nor collect data through observations. Likewise, the interviewers were unable to visit the slaughterhouses and processing plants, and relied on interviewees’ responses, which increased the likelihood of desirability bias.

### Supplementary Information

Below is the link to the electronic supplementary material.

Supplementary file1 (DOCX 21 KB)

Supplementary file2 (DOCX 22 KB)

## Data Availability

Not applicable.

## Footnotes

1 Sustainable development goals: 1 (no poverty), 2 (zero hunger), and 12 (responsible consumption and production).
