# Correction to: Mapping of dressed and processed poultry products in Bangladesh: Identifying the food safety risks for policy intervention

**DOI:** 10.1007/s11259-023-10174-2

**Published:** 2023-07-18

**Authors:** Jinnat Ferdous, Helal Uddin, Rashed Mahmud, Mathew Hennessey, Abdullah Al Sattar, Suman Das Gupta, Justine S. Gibson, Robyn Alders, Joerg Henning, Guillaume Fournié, Ahasanul Hoque

**Affiliations:** 1 School of Veterinary Science, The University of Queensland, Queensland, Australia; 2 Chattogram Veterinary and Animal Sciences University, Chittagong, Bangladesh; 3 Veterinary Epidemiology, Economics and Public Health Group, Department of Pathobiology and Population Sciences, Royal Veterinary College, London, UK; 4 Gulbali Institute, Charles Sturt University, Wagga Wagga, NSW Australia; 5 Development Policy Centre, Australian National University, Canberra, NSW Australia; 6 Global Health Program, Chatham House, London, UK; 7 INRAE, VetAgro Sup, UMR EPIA, Université de Lyon, Marcy l’Etoile, 69280 France; 8 INRAE, VetAgro Sup, UMR EPIA, Université Clermont Auvergne, Saint Genes Champanelle, 63122 France

**Correction to: Veterinary Research Communications**

10.1007/s11259-023-10153-7

In the published paper, there was an error in the address of the affiliation of the fifth co-author Suman Das Gupta. The address of the Gulbali Institute should be read, “Gulbali Institute, Charles Sturt University, Wagga Wagga, NSW, Australia”.

The original articles has been corrected.

