# Supplementary material for: Mapping of dressed and processed poultry products in Bangladesh: Identifying the food safety risks for policy intervention
Source: Vet Res Commun. 2023 Jun 27;47(4):1991–2002. doi: 10.1007/s11259-023-10153-7 (PMC10697896; doi:10.1007/s11259-023-10153-7)
Supplement: Supplementary file 1 — Supplementary file1 (DOCX 21 KB) [file 11259_2023_10153_MOESM1_ESM.docx]

# **Journal name:** Veterinary Research Communication

# **Title:** Mapping of dressed and processed poultry products in Bangladesh: identifying the food safety risks for policy intervention

**Jinnat Ferdous^1^, Md Helal Uddin^2^, Rashed Mahmud^2^, Matthew Hennessey^3^, Abdullah Al Sattar^2^, Suman Das Gupta^1^, Justine S. Gibson^1^, Robyn Alders^4,5^, Joerg Henning^1^, Guillaume Fournié^3^, Md. Ahasanul Hoque^2^**

^1^ School of Veterinary Science, The University of Queensland, Australia

^2^ Chattogram Veterinary and Animal Sciences University, Bangladesh

^3^Veterinary Epidemiology, Economics and Public Health group, Department of Pathobiology and Population Sciences, Royal Veterinary College, UK

^4^Development Policy Centre, Australian National University, Canberra, NSW, Australia

^5^Global Health Program, Chatham House, London, UK

**Corresponding author:** Jinnat Ferdous; [j.ferdous@uq.edu.au](mailto:j.ferdous@uq.edu.au)

**Supplementary Information 1**

*Questionnaire guide for slaughterhouse companies*

| Company name: |
| --- |
| Interviewer’s name: |
| Interviewee name, designation, and email address: |
| Date of interview: |
| Duration of interview: minutes  Time: Start: ; End: |
| Slaughterhouse establishment year: |
| 1. Slaughterhouse |
| - 1. How many slaughterhouses are managed by the company? |
| - 1. Which kind of birds are being slaughtered? |
| - 1. What are the origin of the chickens: farms owned by the company and/or farms contracted by the company? Also independent farms? Other? |
| - 1. If chickens are only provided by company-owned and contracted farms: is the slaughterhouse processing all the chickens produced by those farms? If not, who buys the "excess" chickens (e.g., other companies for their slaughterhouses, middlemen, or market vendors)? |
| - 1. How does it work? Or how does it operate? |
| - 1. Are all birds (Sonali or exotic broiler or spent layer or spent breeder layer or all) slaughtered by halal method? |
| - 1. Manual or automatic slaughtering? |
| - 1. How many birds are slaughtered daily? |
| - 1. What hygienic measures are followed? |
| 1. Quality assurance (Testing for meat. Plant equipment, air, water, etc.) |
| 1. Only Dressed meat or any other products (like a nuggets, sausage, chicken balls etc.) are being produced? |
| 1. Packaging system? |
| 1. Transporting and delivery system |
| - 1. Who transports the birds from the farms to the slaughterhouse: company employees or contracted traders? Types of vehicles?   2. Who transports the chickens from the slaughterhouse to the outlets/customers? Types of vehicles?   3. How is the cold chain maintained?   4. How are vehicles managed (hygiene, biosecurity, etc.)? |
| 1. Personnel involved? |
| 1. How frequently are products transported? |
| 1. Where are products delivered? (Retail? e.g. Customer types, restaurants, fast-food industries, cities, super shops, etc.) |
| 1. Home delivery service: how does it work? WhatsApp, Facebook, dedicated app? |
| 1. Proportion of outputs that goes to "home delivery", company owned shops, other shops, and restaurants? |
| 1. Any quality check at delivery? |
| 1. Wholesale price? Retail price? |
| 1. Daily trading amount? |
| 1. Do you have any effluent treatment plants (ETP) and water treatment plants (WTP)? |
