# Supplementary material for: Mapping of dressed and processed poultry products in Bangladesh: Identifying the food safety risks for policy intervention
Source: Vet Res Commun. 2023 Jun 27;47(4):1991–2002. doi: 10.1007/s11259-023-10153-7 (PMC10697896; doi:10.1007/s11259-023-10153-7)
Supplement: Supplementary file 2 — Supplementary file2 (DOCX 22 KB) [file 11259_2023_10153_MOESM2_ESM.docx]

# **Journal name:** Veterinary Research Communication

# **Title:** Mapping of dressed and processed poultry products in Bangladesh: identifying the food safety risks for policy intervention

**Jinnat Ferdous^1^, Md Helal Uddin^2^, Rashed Mahmud^2^, Matthew Hennessey^3^, Abdullah Al Sattar^2^, Suman Das Gupta^1^, Justine S. Gibson^1^, Robyn Alders^4,5^, Joerg Henning^1^, Guillaume Fournié^3^, Md. Ahasanul Hoque^2^**

^1^ School of Veterinary Science, The University of Queensland, Australia

^2^ Chattogram Veterinary and Animal Sciences University, Bangladesh

^3^Veterinary Epidemiology, Economics and Public Health group, Department of Pathobiology and Population Sciences, Royal Veterinary College, UK

^4^Development Policy Centre, Australian National University, Canberra, NSW, Australia

^5^Global Health Program, Chatham House, London, UK

**Corresponding author:** Jinnat Ferdous; [j.ferdous@uq.edu.au](mailto:j.ferdous@uq.edu.au)

**Supplementary Information 2**

Questionnaire guide for supermarkets

Name of the supermarket:

Outlet address:

Interviewer:

Interviewee:

Date:

Duration of interview: minutes

Time: Start: ; End:

1. Total number(s) of outlets and location?
2. Do you get dressed birds or live birds for selling?
3. If you buy dressed birds for selling-
   1. Type of dressed birds (only broiler/ any other kinds)
   2. Who supplies the dressed birds (company/Live Bird Market/middlemen/own farms)?
   3. What is the buying and selling price (for each source- company/LBM/middlemen/own farms) of each type of bird?

| Species | Buying price (dressed per kg) | Selling price (per kg) |
| --- | --- | --- |
| Broiler with skin from slaughtering company |  |  |
| Broiler without skin from slaughtering company |  |  |
| Sonali |  |  |
| Deshi |  |  |
| Pigeon |  |  |
| Duck |  |  |
| Turkey |  |  |
| Quail |  |  |

- 1. On a typical day, who transports the dressed birds from the source to the outlet?
  2. What types of vehicles are used to transport dressed birds from source to outlet?
  3. If you get dressed chicken from LBM/own farms/middlemen, how do you maintain the cold chain during transportation?
  4. Are there any on-spot quality assurance when you get dressed birds from any poultry company/middlemen/own farms (Describe the testing etc.)?

1. Do you sell live birds in your outlet?
   1. If yes, which type of birds?
2. If live birds are brought for selling-
   1. Which types of live birds are usually brought to the outlet?
   2. Where do you get live birds for your outlets?
   3. What is the buying and selling price (for each source- LBM/ middlemen/own farms) of each type of bird?
   4. Where do you slaughter the birds? e.g. At the source/at the corner of the super shop?
   5. Who slaughters the birds? Do you follow the halal method of slaughtering?
   6. How do you ensure the quality of birds before buying them from the source?
   7. When do you slaughter chickens? i.e., Do you store some chickens live and the timing of slaughter depends on sales?
   8. When birds are brought from your own farms, are there any veterinary ante-mortem and post-mortem examinations done to certify that the products you are selling are safe for consumption?
   9. How do you dispose of poultry waste if you dress them at your point of sale?
3. How do you set the selling price?
4. Is there any difference between wholesale and retail prices? If yes, please explain.
5. On a typical day, how many (in kg) birds are usually brought to this outlet for sale? Specify the amount (in kg) for each type.
6. What is the amount (in kg) of birds sold on a typical day? Specify the amount (in kg) for each type of bird.
7. How frequently do you transport chicken from the source to the outlet?
8. What are the hygienic measures you follow at the super shop? (e.g., washing of the dressed bird, during live birds slaughtering, etc.)
9. How do you maintain the cold chain at your outlet?
10. How do you ensure quality in your outlet? (Cleaning/disinfection etc.; frequency of cleaning)
11. If the quality of any whole chicken or chicken products deteriorates during transport, what do you do with those chicken /products?
12. Do you have any home delivery service? If yes, how does it work?

How do you maintain the cold chain during home delivery services? Do you use the same carrier and vehicle again and again on the same day for home delivery?

1. Do you have leftover chicken on a particular day generally? If yes, do you sell leftover chicken the next day?

How do you decide the expiry date of your products? What is the maximum length of time you keep a dressed chicken in your shop if it remains unsold?

1. Do you supply chicken to hotels/restaurants/hospitals or other institutions? If yes, via middlemen or directly? How do you fix the prices for these institutes?
